# Estimation of aboveground and belowground carbon stocks in urban freshwater wetlands of Sri Lanka

**DOI:** 10.1186/s13021-020-00152-5

**Published:** 2020-09-02

**Authors:** Deekirikewage Dona Thamali Lushanya Dayathilake, Erandathie Lokupitiya, Vithana Pathirannehelage Indika Sandamali Wijeratne

**Affiliations:** 1 University of Colombo, PO Box 1490, Colombo 03, Sri Lanka; 2 Department of Geography, University of Colombo, Colombo 03, Sri Lanka

**Keywords:** Freshwater wetlands, Aboveground and belowground biomass, Tropical carbon stocks

## Abstract

**Background:**

The occurrence of climate change at an unprecedented scale has resulted in alterations of ecosystems around the world. Numerous studies have reported on the potential to slow down climate change through the sequestration of carbon in soil and trees. Freshwater wetlands hold significant potential for climate change mitigation owing to their large capacity to sequester atmospheric carbon dioxide (CO_2_). Wetlands among all terrestrial ecosystems have the highest carbon density and are found to store up to three to five times more carbon than terrestrial forests. The current study was undertaken to quantify carbon stocks of two carbon pools: aboveground biomass (AGB) and belowground biomass (BGB). Chosen study sites; Kolonnawa wetland and Thalawathugoda wetland park are distributed within the Colombo wetland complex. Colombo was recognized as one of the 18 global Ramsar wetland cities in 2018. A combination of field measurements and allometric tree biomass regression models was used in the study. Stratification of the project area was performed using the normalized difference vegetation index (NDVI).

**Results:**

The AGB carbon stock, across strata, is estimated to be in the range of 13.79 ± 3.65–66.49 ± 6.70 tC/ha and 8.13 ± 2.42–52.63 ± 10.00 tC/ha at Kolonnawa wetland and Thalawathugoda wetland park, respectively. The BGB carbon stock is estimated to be in the range of 2.47 ± 0.61–10.12 ± 0.89 tC/ha and 1.56 ± 0.41–8.17 ± 1.39 tC/ha at Kolonnawa wetland and Thalawathugoda wetland park, respectively. The total AGB carbon stock of Kolonnawa wetland was estimated at 19,803 ± 1566 tCO_2_eq and that of Thalawathugoda wetland park was estimated at 4180 ± 729 tCO_2_eq.

**Conclusions:**

In conclusion, the study reveals that tropical freshwater wetlands contain considerable potential as carbon reservoirs. The study suggests the use of tropical freshwater wetlands in carbon sequestration enhancement plans in the tropics. The study also shows that *Annona glabra*, an invasive alien species (IAS), has the potential to enhance the net sink of AGB carbon in these non-mangrove wetlands. However, further studies are essential to confirm if enhanced carbon sequestration by *Annona glabra* is among the unexplored and unreported benefits of the species.

## Background

The occurrence of climate change at an unprecedented scale is indicated by many studies and global assessments. IPCC [1] indicates that global warming is likely to reach 1.5 °C above pre-industrial levels between 2030 and 2052 if existing trends are to persist. Further, the IPCC special report on emissions scenarios projects an increase of 25–90% carbon dioxide equivalent (CO_2_eq) of global greenhouse gas (GHG) emissions between 2000 and 2030 [2]. Carbon estimation across different ecosystems has thus become a prerequisite for establishing carbon sequestration enhancement plans in mitigating climate change.

The extent of world’s wetlands is estimated to be about 5–8% of the total land surface on earth [3]. Despite this low representation as a percentage of area on land, wetlands among all terrestrial ecosystems have the highest carbon density. These ecosystems are found to store up to three to five times more carbon than terrestrial forests [4]. When considering global averages, figures reported for mangroves and marshes generally far exceed those of tropical and temperate forests where estimates have recorded less than 400 tC/ha [5]. However, there is an interesting and a much-debated controversy on the net effect of wetlands on the global carbon budget. Some argue that wetlands occasionally act as carbon sources while many have recognized wetlands as carbon sinks [6]. In fact, carbon dioxide (CO_2_), methane (CH_4_), and, nitrous oxide (N_2_O) are found to be released from wetlands [6]. When viewing wetlands as a source of CO_2_, natural wetlands emit approximately 1.45 × 10^11^ kg CH_4_-C yr^−1^ to the atmosphere which is equivalent to about 25% of the total emissions due to anthropogenic and natural sources [7]. Mitra et al. [8] have gone to a great extent to answer this controversial question. They provide a net balance between CH_4_ production and carbon sequestration for the world’s wetlands and deduce the overall impact of wetlands on climate change to be minimal.

Plant biomass carbon stock, a combination of both AGB carbon and BGB carbon represents an important component of the total carbon stock of an ecosystem. Study sites considered in the present study, Kolonnawa wetland and Thalawathugoda wetland park are distributed within the Colombo wetland complex of the Western province of Sri Lanka. Most parts of the Colombo wetland complex are currently dominated by *Annona glabra,* an IAS [9]. Interestingly, several studies have identified *Annona glabra* as a species showing some atypical growth properties. Flooding has shown to induce significant increments in root, stem, whole-plant biomass, and root: shoot biomass ratio [10]. The same study further suggests enhanced net primary productivity in flooded conditions.

When considering carbon estimation, most existing studies have focused on dry-land ecosystems that extend over large areas and have not accounted for the many, small, and scattered carbon-storing ecosystems such as mangrove swamps and salt marshes [11]. Thus, there is still uncertainty regarding the quantitative contribution of these ecosystems to the global carbon cycle [11]. Khanh and Subasinghe [12] have previosuly estimated the biomass carbon stock of mangrove communities at the Muthurajawela wetland of Sri Lanka at 22.05 tC/ha. Perera and Amarasinghe [13] have estimated the carbon accumulation rate of mangroves at the Negombo estuary of Sri Lanka at 12 t/ha/yr.

Currently, more than 50% of the entire population live in urban areas and this figure is expected to increase beyond 60% by 2050. This urbanization brings about a bigger challenge to make our urban areas sustainable and resilient to disasters including impacts of climate change [14]. A combination of approaches is necessary to make our urban cities sustainable and wetlands play a crucial part in this. Urban wetlands could be understood as those wetlands that have survived rapid urbanization or as wetlands that are newly constructed in urban areas [14]. Interestingly, during the past decade, much focus has been placed on urban wetlands internationally and regionally owing to multiple reasons. Urban wetlands fulfill crucial services for people and the environment. They act as regulators of urban floods while reducing the subsequent infrastructure and economic damage. Further they act as purifiers of urban pollution which includes both water and air purification via filtration [14].

Colombo, the commercial capital and the largest city of Sri Lanka, comprises a complex of man-made lakes, canals, marshes, paddy fields that are abandoned or currently in use [15]. The government of Sri Lanka is ambitious to harness the potential of the Colombo wetland complex to buffer the commercial capital of Sri Lanka against floods and similar events which are expected to increase in the future with climate change. A report outlined in favour of the Colombo wetland complex by the World Bank Climate Change Group and the Global Facility for Disaster Reduction and Recovery has recognized carbon storage as one of the co-benefits of the Colombo wetland complex [16].

The present study was based on the technique of biomass estimation by direct tree measurement, coupled with remote sensing techniques for stratification purposes. The concept of coupling remote-sensing data with field data has been tested and used extensively in the last two decades [17–19]. There is growing evidence suggesting that integration of remote-sensing data with ecological models significantly underpins and enhances the study of ecological processors and environmental variables [17, 18]. In this study, a stratification strategy was adopted to compartmentalize the wetland areas based on the density of vegetation. Stratification was performed using the Landsat surface reflectance-derived NDVI. The NDVI is used to measure the vigor of vegetation on Earth [20]. The parameter that is measured from remotely-sensed NDVI is the fraction of absorbed photosynthetically active radiation by the photosynthesizing tissue in a canopy to incident photosynthetically active radiation – f_APAR,_ (dimensionless) [18]. The NDVI is defined as follows [18]:

1 $$\left( {{\text{NIR }}{-}{\text{ Red}}} \right)/\left( {{\text{NIR}}\, + \,{\text{Red}}} \right)$$

where NIR is the spectral radiation from a near-infrared band and Red is the spectral radiation from a red band [18]. The accuracy of stratification depends, for example, on how well the field observations could be linked to the NDVI images derived from Landsat imagery.

In this study, allometric biomass models were used to quantify AGB and BGB. Allomteric biomass models are constructed using regression analysis based on functional relationships between tree biomass and tree dimensions such as stem diameter, tree height and wood density [21]. The plot biomass was calculated by measuring the tree dimensions of individual trees in the plot and calculating the tree biomass using allometric biomass models [21].

The primary objective of the current study was to estimate the AGB and BGB carbon stocks of two major freshwater wetlands, Kolonnawa wetland and Thalawatugoda wetland park, located within the Colombo wetland complex. Secondarily, the study aimed to establish a comparison among AGB and BGB stocks of various ecosystems to better assess the relative potential of freshwater wetlands to store carbon. The study sites were distributed within a Ramasar Wetland City. In 2018, a total of eighteen cities worldwide were awarded the wetland city accreditation by the Ramsar Convention and Colombo was among the accredited cities [22]. Thus, the present study was designed to cater to an important knowledge gap as no carbon estimates have been documented for these wetlands distributed within the Ramsar Wetland City. The results hold much significance as the amount of carbon sequestrated or released from tropical freshwater wetlands has been poorly quantified.

## Results

The analysis was conducted separately for the two different study sites, Kolonnawa wetland and Thalawathugoda wetland park. This section will initially present results of the NDVI classification. It will be followed by a summary of the inventoried species composition and estimates of AGB and BGB.

### Sampling plots determined via stratified random sampling

**Fig. 1:**
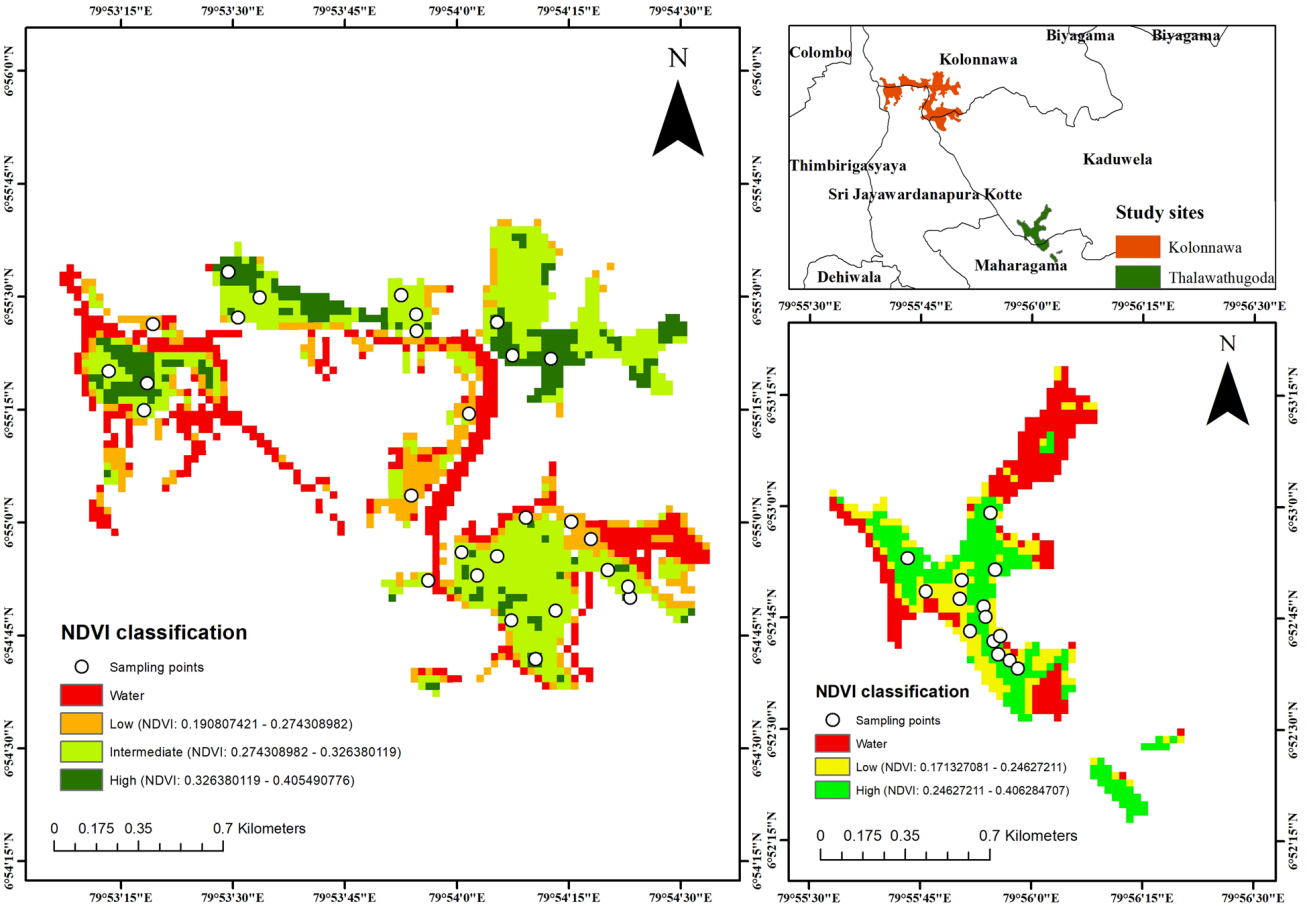
Kolonnawa wetlandand Thalawathugoda wetland park stratified into strata according to the NDVI. Kolonnawa wetland was stratified into four strata out of which three strata represent vegetation and Thalawathugoda wetland park was stratified into three strata out of which two strata represent vegetation. The inset in the upper right shows the location of the study area within the a section of the Colombo wetland complex

Kolonnawa wetland and Thalawathugoda wetland park were categorized into three strata according to the NDVI classes. The strata are detailed in Table 1. Figure 1 presents the results of the stratification performed for the two study sites based on the NDVI.

**Table 1:** Summary of strata of each study site

Study site	Stratum number	NDVI range	Stratum name	Stratum area (ha)	Number of plots
Kolonnawa wetland	0	− 0.02537907 – 0.190807421	–	NA	NA
	1	0.190807421 – 0.286824791	1	31.82	7
	2	0.286824791 – 0.326380119	2	53.98	14
	3	0.326380119 – 0.405490776	3	21.48	7
	Total			107.28	28
Thalawathugoda wetland park	0	− 0.05879659 – 0.171327081	–	NA	NA
	1	0.171327081 – 0.24627211	1	13.28	6
	2	0.24627211–0.353396207	2	19.59	8
	Total			32.87	14

Classification was based on the NDVI. The table presents the area of each stratum (ha) and the number of

10 m radius plots in which sampling was carried out along with the NDVI range for each stratum

Stratum 0 has not been considered as it represents water bodies

### Number of inventoried trees

A total number of 2703 and 629 trees were inventoried at Kolonnawa wetland and Thalawathugoda wetland park, respectively. *Annona glabra* represent 90% of the total inventoried trees belonging to 17 species at Kolonnawa wetland. Other species with an abundance of 1% or more included *Cerbera odollam*, *Morinda citrifolia* and *Syzygium cumini.* Fifteen species were inventoried at Thalawathugoda wetland park. The park owns a rich diversity of shrub species and small woody species of diameter at breast height (DBH) < 5 cm. These shrub and small woody species were not inventoried in the study due to their insignificant contribution to the overall carbon stock. Those species with an abundance above 1% included *Annona glabra*, *Macaranga peltata*, *Trema oreintalis* and *Syzygium caryophyllatum*.

### Distribution of diameter at breast height

**Fig. 2:**
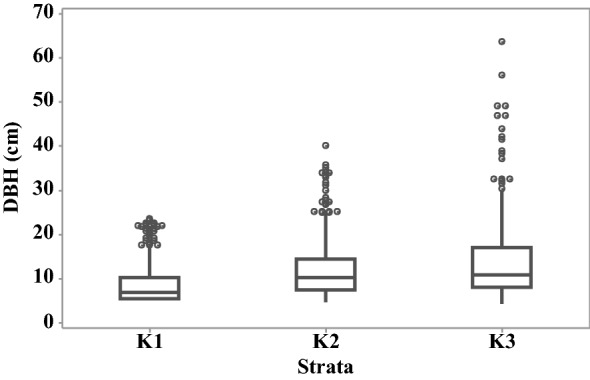
Distribution of diameter at breast height (cm) across strata at Kolonnawa wetland. Individual circular points represent outliers

**Fig. 3:**
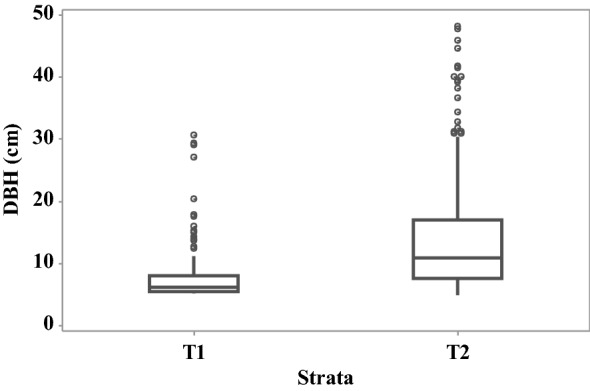
Distribution of diameter at breast height (cm) across strata at Thalawathugoda wetland park. Individual circular points represent outliers

Level of variation of DBH at each stratum at Kolonnawa wetland and Thalawathugoda wetland park are given in Fig. 2 and Fig. 3, respectively. Level of variation of DBH along strata was observed to be increasing from stratum 1 to stratum 3.

### Estimates of aboveground biomass and belowground biomass

Table 2 presents the estimates of AGB and BGB obtained via allometric models for Kolonnawa wetland and Thalawathugoda wetland park. The uncertainty was relatively higher, in all cases, for stratum 2 and 3, corresponding with strata having higher variability in DBH at Kolonnawa wetland. In the case of Thalawathugoda wetland park the uncertainty was higher, in all cases, for stratum 2 corresponding with the stratum having higher variability in DBH.

**Table 2:** Estimates of average AGB—aboveground biomass (t/ha) and BGB—belowground biomass (t/ha)

	Stratum	AGB (t/ha)	BGB (t/ha)
Kolonnawa wetland	1	27.57 ± 7.30	6.33 ± 1.56
	2	132.97 ± 13.40	25.94 ± 2.28
	3	127.36 ± 18.10	24.98 ± 3.13
Thalawathugoda wetland park	1	16.25 ± 4.84	3.99 ± 1.05
	2	105.26 ± 20.00	20.96 ± 3.56

### Estimates of ecosystem level carbon stocks

The carbon content was considered 50% of the biomass. Table 3 below presents the average estimates of AGB and BGB carbon at Kolonnawa wetland and Thalawathugoda wetland park. The total AGB carbon stock of Kolonnawa wetland and Thalawathugoda wetland park was estimated at 19,803 ± 1566 tCO_2_eq and 4,180 ± 729 tCO_2_eq, respectively.

**Table 3:** Estimates of mean stratum carbon (t/ha), total stratum carbon (t) and total ecosystem carbon (t)

Stratum	Carbon Pool	Mean Stratum C (t/ha)	Total Stratum C (t)	Total Aboveground C (t)	Total Belowground C (t)
Kolonnawa wetland					
1	Aboveground	13.79 ± 3.65	438.80 ± 116.14		
	Belowground	2.47 ± 0.61	78.60 ± 19.41		
2	Aboveground	66.49 ± 6.70	3 589.13 ± 361.67		
	Belowground	10.12 ± 0.89	546.28 ± 48.04		
3	Aboveground	63.68 ± 9.05	1 367.85 ± 194.39		
	Belowground	9.74 ± 1.22	209.22 ± 26.21		
				5 395.78 ± 426.71	834.10 ± 58.07
Thalawathugoda wetland park					
1	Aboveground	8.13 ± 2.42	107.97 ± 32.14		
	Belowground	1.56 ± 0.41	20.72 ± 5.44		
2	Aboveground	52.63 ±10.00	1 031.02 ± 195.90		
	Belowground	8.17 ± 1.39	160.05 ± 27.23		
				1 138.99 ± 198.52	180.77 ± 27.77

## Discussion

This study seeks to present an estimate of the total aboveground and belowground carbon stock of two urban freshwater wetlands; Kolonnawa wetland and Thalawathugoda wetland park both of which are located within the Colombo city, the commercial capital of Sri Lanka. Colombo city was declared as a Ramsar wetland city in 2018 at the 13^th^ Conference of the Parties to the Ramsar Convention on Wetlands.

### Estimated carbon storage potential

The high potential of the freshwater wetlands under study in terms of carbon storage was made evident by the significant concentrations of both AGB and BGB carbon. Average AGB carbon stock of Kolonnawa marsh, 13.79 ± 3.65–66.49 ± 6.70 tC/ha (across strata) is comparable with previous estimates published for mangrove communities at the Muthurajwela wetland (i.e., 22.05 tC/ha; [12]) and lower than previosuly published figures for mangroves at the Batticaloa lagoon, Sri Lanka (i.e., 246 t/ha equivalent to 123 tC/ha; [23]) and that of the Negombo estuary, Sri Lanka (i.e., 163.72 t/ha equivalent to 81.86 tC/ha; [13]). According to Verwer and Van der Meer [24], estimates of AGB in tropical peat swamp forests lie within a range of 132–199 t/ha (equivalent to 66–100 tC/ha). Thus, the AGB stocks at Kolonnawa wetland were comparable with the AGB carbon stocks of tropical peat swamp forests.

The AGB carbon stocks of the freshwater wetlands of the present study (13.79 ± 3.65–66.49 ± 6.70 tC/ha) were lower compared to tropical wet zone forests (249 tC/ha [25], 237.2 tC/ha; [26]), and dry zone forests (77 tC/ha; [25]) although they have higher biomass stocks compared to dry zone homegardens (3.31 tC/ha; [27]). However, BGB carbon stocks found in the current study (2.47 ± 0.61–10.12 ± 0.89 tC/ha) seem to be lower than the previously reported values from certain other studies; peat swamps of Encrucijada Biosphere Reserve of Mexico, a tropical riverine wetland (43.5 t/ha equivalent to 16.97 tC/ha; [28]) and Pekan Pahang, Malaysia, a peat swamp forest (69.48 t/ha equivalent to 27.10 tC/ha; [29]), suggesting potential higher rates of decomposition or lower litter density in our wetlands, which need further investigation.

### Role of invasive alien species in carbon storage

Biomass carbon stocks of Kolonnawa wetland which has a denser population of *Annona glabra*, an IAS, are higher compared to Thalawathugoda wetland park. Perhaps, the high density and DBH levels of *Annona glabra* seem to play a significant role in enhancing the carbon storage in these non-mangrove wetlands. Traditionally IASs are viewed as ecosystem invaders which disrupt native biodiversity. They often disrupt the existing ecosystem structure and function. However, there is an interesting shift of opinion among scientists on IASs in the context of carbon sequestration. Some scientists view IASs as excellent carbon sinks; particularly in the case of blue carbon [30]. The present study supports the above claim, as higher carbon stocks are reported from Kolonnawa wetland in comparison to Thalawathugoda wetland park. However, further studies that view these species in all dimensions are essential to confirm if carbon sequestration by IASs is among the unexplored and unreported benefits of *Annona glabra*. This is of particular importance as *Annona glabra* has been identified as a significant threat to native ecological character and native biodiversity within the Colombo wetland complex [15]. Further, the spread of *Annona glabra* is of particular concern as it has the ability to transform wetlands into terrestrial ecosystems by enhancing the process of natural succession of wetlands [31]. In fact, most parts of Kolonnawa wetland are currently dominated by monospecific stands of *Annona glabra*.

### Ecosystem services and overall importance of the Colombo wetland complex

A substantial number of published studies demonstrate that the Colombo wetland complex deliver a great variety of benefits [9, 15, 32, 33]. A study by Mcinnes and Everard [32] has assessed the benefits provided by the wetlands distributed within Colombo in 62 different wetland sites. The study reports 35 diverse ecosystem services distributed across a range of scales, from local through regional to global scale. According to Mcinnes and Everard [32], the provision of habitat is identified as the ecosystem service that has the highest positive contribution. The same study has identified regulation of water; flood water attenuation and storage [9], regulation of global climate and photosynthesis as significant ecosystem services performed by these wetlands [32]. Interestingly, some studies have pointed out that these wetlands aid in reducing thermal discomfort and mitigating the urban heat island effect in their respective urban areas [34, 35].

Despite the increasing understanding of the range of benefits provided by the Colombo wetlands, the wetlands are experiencing an accelerated loss and degradation as a result of urban development [9, 33]. In fact, when considering Kolonnawa marsh, the rate of conversion from wetland to non-wetland areas has been as high as 65% between 1981 and 2008 [33].

Perhaps, the figures presented by the current study for biomass carbon stocks at Kolonnawa wetland and Thalawathugoda wetland have the ability to broaden the discussion of conservation around the importance of Colombo wetlands in terms of their contribution towards mitigating climate change. These wetlands could potentially be used in carbon offset programmes which would in turn ensure the conservation of these invaluable ecosystems. It is vital that funds are allocated nationally and globally in the direction of conservation and maintenance of these freshwater wetlands.

## Conclusions

The AGB carbon stock is estimated to be in the range of 13.79 ± 3.65–66.49 ± 6.70 tC/ha and 8.13 ± 2.42–52.63 ± 10.00 tC/ha at Kolonnawa wetland and Thalawathugoda wetland park, respectively. BGB carbon stock is estimated to be in the range of 2.47 ± 0.61–10.12 ± 0.89 tC/ha and 1.56 ± 0.41–8.17 ± 1.39 tC/ha at Kolonnawa wetland and Thalawathugoda wetland park, respectively. The results of the study show that *Annona glabra*, an IAS, has a high capacity to store carbon. However, further studies are required to verify if enhanced carbon sequestration by *Annona glabra* is among the unexplored and unreported benefits of the species. Overall, the study proposes freshwater wetlands as a pragmatic solution for the increasing concentrations of atmospheric CO_2_ and thus suggests the optimization of these ecosystems for the enhancement of the net tropical carbon sink. Further, the study contributes to the body of knowledge on the potential of tropical freshwater wetlands to act as regulators of global climate by influencing the carbon cycle via carbon sequestration.

## Method

The present study is based on a combination of techniques; the technique of estimation by direct measurement coupled with remote sensing techniques for stratification purposes. Field procedures were based on commonly accepted forest inventory practices consistent with the guidelines provided by the IPCC [5].

### Study area

**Fig. 4:**
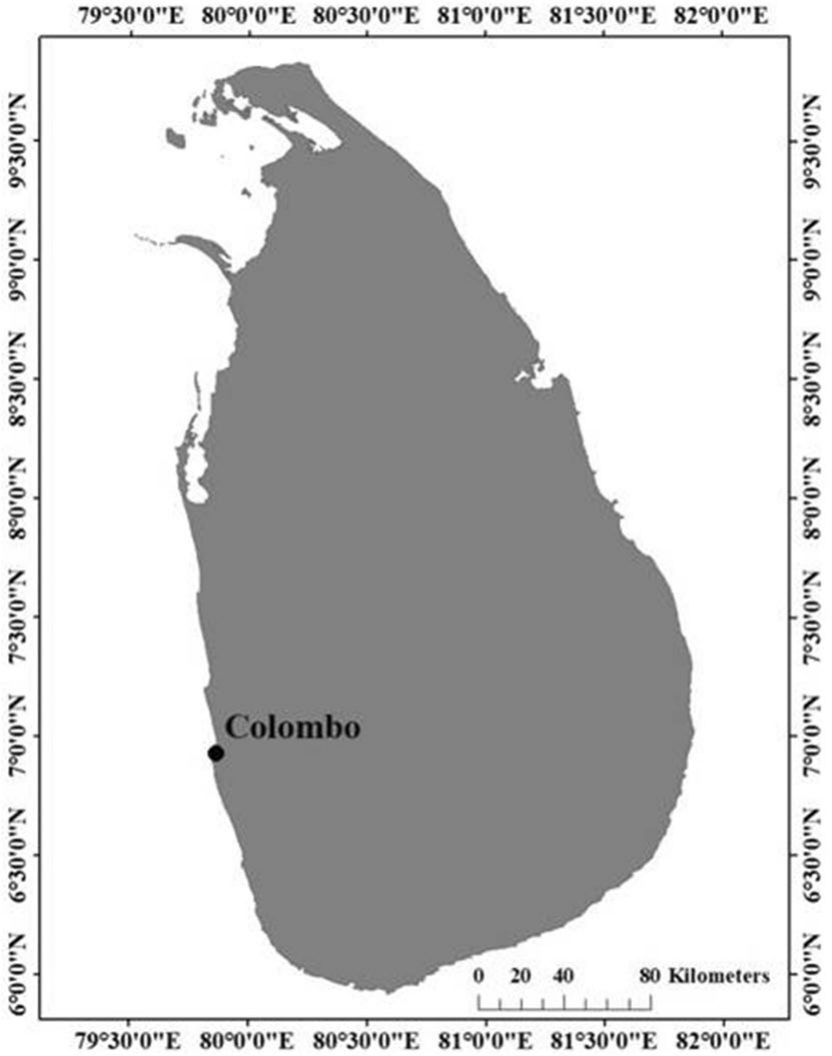
Map showing the location of Colombo, the commercial capital of Sri Lanka

**Fig. 5:**
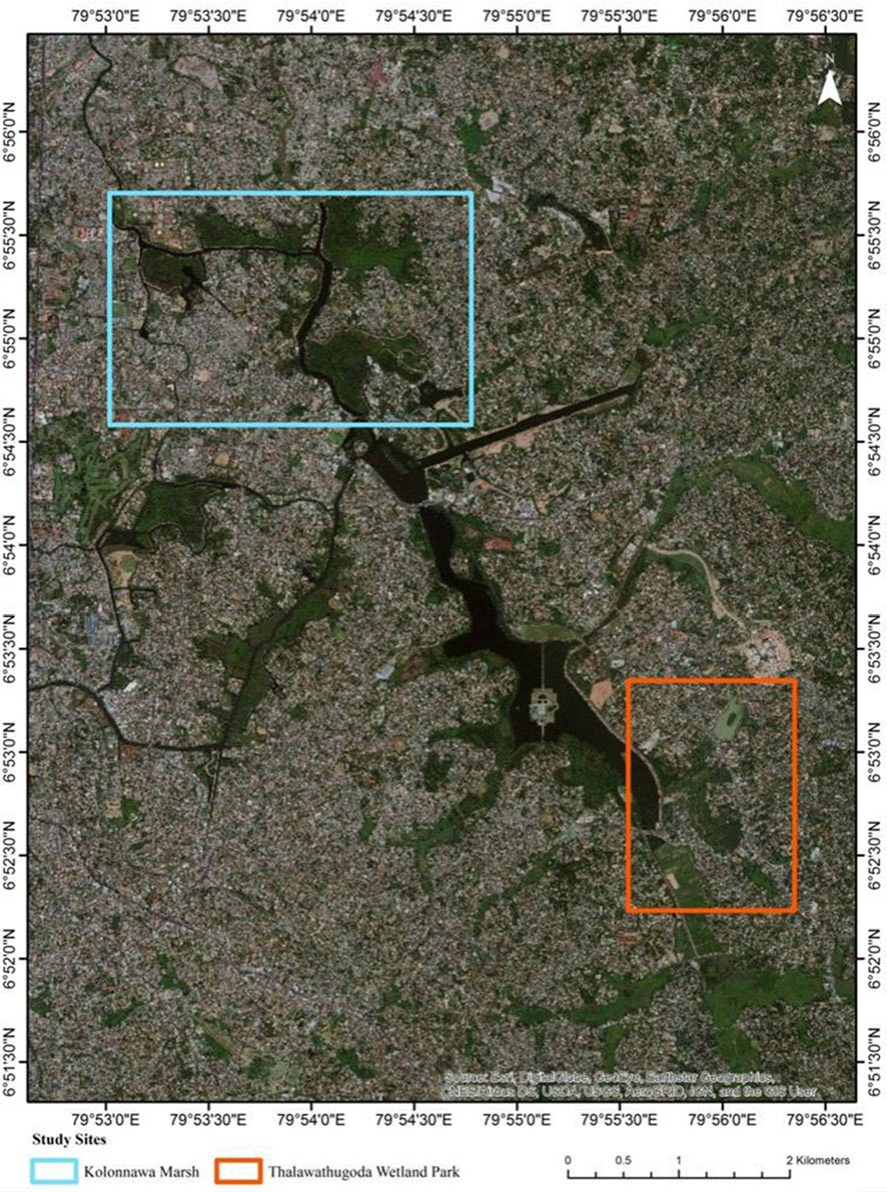
Map showing the study sites

Colombo, the commercial capital of Sri Lanka, accredited a Ramsar wetland city during the 13th Conference of the Parties to the Ramsar Convention on Wetlands in 2018, comprises an assortment of man-made lakes, canals, marshes and paddy fields. Figure 4 shows the location of Colombo on the map of Sri Lanka. The study sites, shown in Fig. 5, Kolonnawa wetland and Thalawathugoda wetland park are distributed within the Colombo Ramsar city and are classified as forested peatlands according to the Ramsar classification of wetlands [15]. The total extents of Kolonnawa wetland and Thalawathugoda wetland park are 107.28 ha and 32.87 ha, respectively.

Hydrology of the two wetlands differs only in a few aspects. Both wetlands undergo seasonal flooding. Perhaps, the surface water levels reach a peak hydro period during the months of May and November. Most parts of the Colombo wetland complex are currently dominated by *Annona glabra*, an IAS [9]. It spreads across urban freshwater wetlands of Sri Lanka, particularly in the Western Province, forming mono-carpets of the single species. The marshes lie within the wet zone and receive a mean annual rainfall of 2,000 mm. The average annual temperature ranges between 25 and 27 °C [36]. The water quality in the Colombo wetlands is severely degraded. A water quality analysis performed against the standards within the European Union’s Water Framework Directive reports the water quality to be bad or very bad in 64% of the wetland areas of the Colombo wetland complex. Domestic waste water represents a significant cause for the degradation of water quality [15].

### Stratification of the project area

Stratification of the project area was performed using Landsat satellite imagery for reasons of efficiency and accuracy of estimations. Landsat 8 images were downloaded from the United States Geological Survey [20]. ArcGIS software was used in analyzing spatial data including the computation of the NDVI. The NDVI values ranged from -1 to 1 where negative values indicate areas without vegetation, values close to zero indicate low density vegetation, and values close to 1 indicate high density vegetation [37]. The NDVI was calculated using the band combination of band 4 (red) and band 5 (near infrared; NIR).

Subsequent adjustments to the default classification were performed via ground truthing to better fit strata to particular vegetation communities. Accordingly, the wetlands were divided into four strata out of which three strata represent vegetation and the other represents the water body. The study only takes into consideration strata representing vegetation.

### Plot location, size, and shape

Field sampling was conducted from July to October 2018. A total of 28 plots of 10 m radius at Kolonnawa wetland and 14 plots of the same radius at Thalawathugoda wetland park were studied. The number of sampling plots per stratum was determined based on the extents of the wetland areas, accessibility of the sites and feasibility of sampling. Only limited sampling could be performed in certain areas due to inaccessibility. The total number of plots was statistically divided among respective strata in a way that the number of plots is proportional to the relative area weight (RAW) of each stratum. RAW was determined by dividing the area of each stratum by the total area of the wetland.

Random sampling plots within each stratum were generated using the ArcGIS software out of which a few plots were shifted deliberately due to difficulties in approaching some of the predetermined locations. Circular plots of radius 10 m were demarcated using a rope. Low perimeter to area ratio, in circular plots, makes it the least vulnerable to errors which could arise due to omission or inclusion of trees at the boundary [38]. Furthermore, Timothy et al. [39] shows that plots of 10 m radius provide a reasonable balance of effort and precision for stem diameters between 20 and 50 cm. Plot locations were recorded using GPS coordinates obtained from the eXplorist510 GPS device.

### Estimation of aboveground biomass

Estimation of ecosystem level AGB was carried out under the steps described by Chave et al. [11] with the inclusion of a few amendments: (1) application of an allometric biomass model for the estimation of individual tree biomass, (2) the summation of AGB across all individual trees at the plot to estimate plot level AGB, (3) calculation of an across-plot average for each stratum to produce a stratum level estimate by the extrapolation of the resultant average into the respective area of each stratum, and 4) the summation of individual stratum level estimate to produce a landscape level estimate.

### Measurement of trees for height and diameter at breast height

All woody tress with a minimum of 5 cm DBH; 5 cm or a larger diameter at the height of 1.3 m within the plot area were considered. With regard to the edge of the plot area only those trees which had 50% of its trunk inside the plot were considered. DBH was measured using a DBH tape at the point of measurement (POM) which was determined using a pole of height 1.3 m. Special considerations were made in the case of forked trees, trees on slopes, and trees with irregularities at the breast height according to UNFCCC [38]. The datasets supporting the results of this article are included within Additional files 1 and 2.

A Suunto clinometer PM5/360 was used for trees with a height greater than 4 m. The horizontal distance from the viewer to the tree was measured using a metric tape. In the case of tress below 4 m, the height was obtained using a graduated pole of 4 m.

### Allometric model selection

Allomteric equations that directly convert stem diameter and sometimes height and wood density to total tree biomass were used in the study (Table 4). In those cases where stem biomass equations were used, biomass expansion factors (BEF) of 22% and 2% for branches and leaves, respectively, were used to convert the stem biomass to the total tree biomass [40].

**Table 4:** Allometric equations used to calculate aboveground and belowground biomass (t) of trees

Allometric equation for AGB	Source	Species
${AGB = 0.1637(DBH)}^{2.2864}$^*a*^	[12]	*Annona glabra*
$AGB= {0.1466(DBH)}^{2.3369}$^*b*^	[12]	*Sonneratia caseolaris, Barringtonia asiatica, Carallia brachiata, Cerbera odollam*
${AGB}_{stem}=0.092486\times \left(DBH\right)\times {H}^{1.4765}$	[40]	*Acacia auriculiformis*
$ABG=\mathit{exp}\left\{-2.4090+0.9522\mathit{ln}\left({DBH}^{2}\times H\times \rho \right)\right\}$	[41]	Other species

*AGB* aboveground biomass, *BGB* belowground biomass, *DBH* diameter at breast height, *H* height, $\rho$ Wood density. Wood density (g/cm^3^) values used for calculating biomass were obtained mainly from [42] and [43]. ^a^ and ^b^ have been developed for green biomass. Thus, green biomass was converted to dry biomass using biomass conversion factors—0.529 and 0.539 for corresponding equations ^a^ and ^b^ according to Khanh and Subasinghe [12]

The BGB of woody tree species was determined using allometric equations presented in [44]. The application of allometric relationships is the most practical and cost effective option for BGB. The chosen model (Eq. 2) has been developed using the root to shoot ratio for tropical tree species.

2 $$BBD = \exp \left( { - 1.0587 + 0.8836{\text{~}} \times lnABD} \right)$$

*where: *BBD = belowground biomass density (t/ha), ABD = aboveground biomass density (t/ha)

### Analysis of aboveground biomass and belowground biomass and corresponding carbon stocks

Plant biomass carbon pools, AGB and BGB per plot were calculated separately to obtain a per plot biomass stock (kg/per plot) which was then converted into AGB and BGB per hectare (t/ha). Mean AGB per hectare and BGB per hectare, per stratum were calculated by averaging the plot level results across all plots in a given stratum. The result thus produced was extrapolated to the total area (ha) of the respective stratum to generate AGB and BGB per stratum of each study site following which corresponding results were combined separately for both AGB and BGB to produce the total AGB and BGB for each study site. Carbon stock of each wetland was calculated by multiplying the biomass stock by a factor of 0.50 for aboveground and 0.39 for belowground biomass [45]. The total carbon density or total carbon stock was converted to CO_2_eq by multiplying carbon stock by 3.67 [45]. The biomass at the individual tree level and the plot level was computed using the python software. Further calculations were performed on the Minitab (18 version) statistical software.

## Supplementary information

**Additional file 1.** Primary data collected from Kolonnawa wetland. This database contains data that were used in the study entitled “Estimation of aboveground and belowground carbon stocks in urban freshwater wetlands of Sri Lanka” The database particularly contains data obtained from Kolonnawa wetland which is one of the study sites included in the study. Each sheet contains data belonging to 7 fields: Local name of the species (Sinhalese name), scientific name, species ID (as assigned by the authors), wood density, height, diameter at breast height (DBH) and biomass of the tree. Wood density values used for calculating biomass were obtained mainly from [43] and [44].

**Additional file 2.** Primary data collected from Thalawathugoda wetland park. This database contains data that were used in the study - Estimation of aboveground and belowground carbon stocks in urban freshwater wetlands of Sri Lanka. The database particularly contains data obtained from Thalawathugoda wetland park which is one of the study sites included in the study. Each sheet contains data belonging to 7 fields: Local name of the species (Sinhalese name), scientific name, species ID (as assigned by the authors), wood density, height, diameter at breast height (DBH) and biomass of the tree. Wood density values used for calculating biomass were obtained mainly from [43] and [44].

## Data Availability

The datasets supporting the conclusions of this article are included as additional files.

## Abbreviations

ABD: Aboveground biomass density
AGB: Aboveground biomass
BBD: Belowground biomass density
BEF: Biomass expansion factors
BGB: Belowground biomass
CH_4_: Methane
CO_2_: Carbon dioxide
CO_2_eq: Carbon dioxide equivalent
DBH: Diameter at breast height
GHG: Greenhouse gas
IAS: Invasive alien species
N_2_O: Nitrous oxide
NDVI: Normalized difference vegetation index
NIR: Near infrared
POM: Point of measurement
RAW: Relative area weight
